# Infection, inflammation and exercise in cystic fibrosis

**DOI:** 10.1186/1465-9921-14-32

**Published:** 2013-03-06

**Authors:** Pauline Barbera van de Weert-van Leeuwen, Hubertus Gerardus Maria Arets, Cornelis Korstiaan van der Ent, Jeffrey Matthijn Beekman

**Affiliations:** 1 Department of Paediatric Pulmonology, Cystic Fibrosis Centre, University Medical Centre Utrecht, Utrecht, the Netherlands; 2 Department of Immunology, University Medical Center Utrecht, Utrecht, The Netherlands; 3 Centre for Molecular and Cellular Intervention, University Medical Center Utrecht, Utrecht, The Netherlands

**Keywords:** Cytokines, Exercise, Immune system, Infection, Inflammation, Lung disease, Respiratory system

## Abstract

Regular exercise is positively associated with health. It has also been suggested to exert anti-inflammatory effects. In healthy subjects, a single exercise session results in immune cell activation, which is characterized by production of immune modulatory peptides (e.g. IL-6, IL-8), a leukocytosis and enhanced immune cell functions. Upon cessation of exercise, immune activation is followed by a tolerizing phase, characterized by a reduced responsiveness of immune cells. Regular exercise of moderate intensity and duration has been shown to exert anti-inflammatory effects and is associated with a reduced disease incidence and viral infection susceptibility. Specific exercise programs may therefore be used to modify the course of chronic inflammatory and infectious diseases such as cystic fibrosis (CF).

Patients with CF suffer from severe and chronic pulmonary infections and inflammation, leading to obstructive and restrictive pulmonary disease, exercise intolerance and muscle cachexia. Inflammation is characterized by a hyper-inflammatory phenotype. Patients are encouraged to engage in exercise programs to maintain physical fitness, quality of life, pulmonary function and health.

In this review, we present an overview of available literature describing the association between regular exercise, inflammation and infection susceptibility and discuss the implications of these observations for prevention and treatment of inflammation and infection susceptibility in patients with CF.

## Introduction

Physical fitness is correlated with life expectancy in an intensity-dependent fashion, both in health and disease [1,2]. Regular exercise is important for preserving muscle strength and function, cardiorespiratory fitness and quality of life [1,2]. To promote and maintain health, healthy adults are encouraged to engage in aerobic exercises of moderate intensity for a minimum of 30 minutes a day, 5 days a week, or vigorous aerobic exercises for a minimum of 20 minutes a day, 3 days a week [2]. Moderate aerobic exercise is defined as exercise, which noticeably accelerates heart rate and requires 3.0 to 6.0 metabolic equivalents (METs) (e.g. slow cycling, brisk walking or swimming), whereas vigorous aerobic exercise requires more than 6.0 METs and causes rapid breathing and a substantial increase in heart rate (e.g. running fast, swimming laps, singles tennis) [2]. Since the intensity of an exercise depends on an individual’s previous exercise experience and their relative level of fitness [2], these exercise prescriptions might differ in intensity for patients with a chronic disease.

Exercise has been described to affect disease incidence by modulating the immune system [1,3-6]. The immune modulatory effects of exercise and the underlying mechanisms remain however poorly studied [1,3-6]. Inflammatory responses appear to depend on the duration and intensity of the exercises [1,3-6]. In healthy individuals regular aerobic exercise of moderate intensity and duration is associated with reduced disease incidences including metabolic diseases (e.g. obesity, type II diabetes mellitus), pulmonary diseases (e.g. asthma, chronic obstructive diseases), infectious diseases (e.g. viral upper respiratory tract infections), certain cancers (e.g. breast, colon, prostate cancer) and musculoskeletal disorders (e.g. rheumatoid arthritis) [1,4]. Furthermore, regular aerobic exercise has been shown to limit inflammation in diseases associated with low-grade inflammation (e.g. obesity, chronic heart failure, atherosclerosis, diabetes) [1,3,4,6-10]. The opposite effect has been observed for vigorous exercise [1,3,4,6-10]. These studies highlight that specific exercise programs may be used to modify the course of chronic inflammatory diseases.

Patients with cystic fibrosis (CF) suffer from chronic infections and severe inflammation, which lead to progressive pulmonary disease [11-13]. CF is caused by genetic mutations in the Cystic Fibrosis Transmembrane conductance Regulator (*CFTR*) gene that encodes for an ATP-regulated ion-channel, which is expressed in many tissues [11-13]. Patients with CF suffer from a reduced exercise capacity, of which pulmonary function, nutritional status and chronic inflammation are important determinants [14-20]. Thus far, no curative therapy is available for CF disease and infections and inflammation are controlled by antibiotics and occasionally immune suppressive drugs [11,12].

In this review, we present an overview of available literature describing the association between exercise, inflammation and infection susceptibility. Over the past years, novel molecular pathways have been uncovered that may connect exercise to immune modulation. We discuss how these findings may be related to patients with CF who are encouraged to engage in physical exercises to maintain physical fitness, quality of life, sputum clearance, pulmonary function and health [21]. Fine-tuning of prescribed exercise programs in patients with CF with regard to exercise intensity, intervals, volume and timing (disease state) in relation to exercise-induced inflammatory responses may further improve quality of life, physical fitness and life expectancy of patients with CF.

### Exercise-related immunological responses in healthy individuals

#### Immune activation by a single exercise session

A single exercise session induces a transient increase in circulating leukocytes numbers, which is dependent on the intensity and duration of the exercise session [4]. Leukocytes are likely mobilized from the marginal pools such as the spleen, lymphatics and blood vessel walls [4]. Recently, it was shown in a rat model that exercise also recruits immune cells from peripheral tissues such as lung and skeletal muscle [22]. Both innate and adaptive immune cells in peripheral blood acquire an activated phenotype during exercise. This is indicated by increased percentages of inflammatory CD14^+^CD16^+^ monocytes, CD56^bright^ NK cells, activated T lymphocytes as well as memory B cells and plasma cells [4]. This may either represent direct activation by immune stimulatory factors or selective recruitment of activated cells towards peripheral blood [4].

A single exercise session also induces a systemic release of immune modulatory peptides that is dominated by interleukin (IL)-6 and followed by a less marked increase of other cytokines, such as IL-10, IL-8 and the IL-1 receptor antagonist (IL-1ra) [7,23]. Cellular sources for these cytokines are immune cells, fat tissue (adipokines), the liver [7,23,24] and skeletal muscles (myokines) [23,25].

The soluble mediators released during a single exercise session play a role in energy metabolism [19], but also impact the transcriptional profile of leukocytes [26-28]. Exercise-associated inflammatory monocytes show increased expression of toll-like receptor (TLR)-2 and −4 [4,29]. Furthermore, post-exercise, increased gene expression of mediators (e.g. IL-6, IL-8) and receptors (e.g. IL-6 receptor antagonist, CD14, IL-17 receptor, Fc receptors such as CD32 and CD16) was observed in leukocytes, whereas other receptors were found to be down-regulated (major histocompatibility complex I and II) [26-28]. Collectively, these profiles indicate that leukocytes are intrinsically altered by exercise, which may lead to changes in innate and adaptive immune effector mechanisms [26-28].

A single exercise session is associated with improved innate immune functions, indicated by an increased phagocytic capacity and production of reactive oxygen species by peripheral blood neutrophils and monocytes [4]. Whether these changes result from preferential recruitment of activated cell populations into peripheral blood or due to intrinsic changes remains questionable [4]. Furthermore, exercise is associated with changes in adaptive immunity, indicated by changes in immunoglobulin levels. A modest increase in salivary immunoglobulin A (sIgA) and peripheral blood IgM levels is observed independent of changes in B and T lymphocyte levels [4]. High sIgA levels are associated with a lower upper respiratory tract infection (URTI) frequency [4]. Other antibodies, such as peripheral blood IgG, show contrary results upon an exercise stimulation [4].

**Figure 1:**
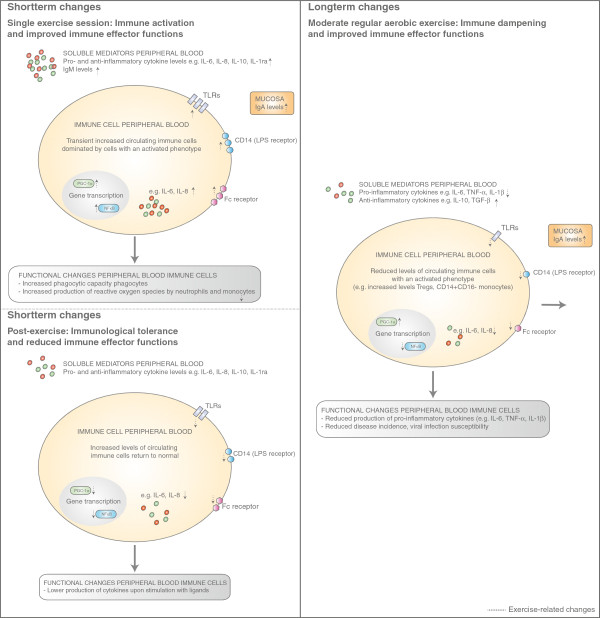
**Exercise**-**related immune responses.** A single exercise session leads to immune activation and improved immune effector functions (left, upper panel), indicated by enhanced circulating levels of immune cells with an activated phenotype and enhanced production of immune modulatory peptides. Upon cessation of the exercise session, immune activation is followed by a tolerizing phase, which is characterized by reduced immune effector functions (left, lower panel). Immune cell levels return to normal or even below normal when the exercise session has been of high intensity and/ or duration. Immune cell responsiveness is reduced, indicated by a diminished production of immune modulatory peptides upon stimulation with ligands. Long-term changes induced by regular exercise result in immune dampening and improved immune effector functions, indicated by a reduced disease incidence and infection susceptibility (right).

In summary, these results show that a single exercise session induces immune system activation. It evokes a leukocytosis, dominated by cell subsets with an activated phenotype, and the release of mediators such as cytokines and chemokines. These may impact immune effector functions, such as defence mechanisms against pathogens, by direct activation or indirectly by modulation of gene expression (Figure 1, left upper panel).

#### Post-exercise immunological tolerance

Leukocyte and cytokine levels return to normal within a few hours after terminating the exercise session [4]. Intense exercise results in higher peripheral blood leukocyte and cytokine levels, but is also associated with a reduction of leukocyte numbers after cessation of the exercise session [4]. During this resolution phase, the threshold for immune activation appears to be higher [3,4,7,30]. It has been observed that toll-like receptors (TLR) -1, -2, -3 and −4 and human leukocyte antigens (HLA)-DR expression were reduced in monocytes [3,30]. Furthermore, other reports show reduced phagocytosis by neutrophils [4], lower TLR-induced cytokine production [3,7] and reduced MHC II expression and antigen presentation capacity in mouse macrophages post-exercise [3].

Immune tolerance has also been observed after systemic immune activation by trauma, sepsis or surgery. It is likely caused by mediators, such as catecholamines, which are released during stress [31-34]. In vitro pre-incubation of immune cells with β-adrenergic agonists has been found to suppress LPS induced production of pro-inflammatory cytokines by macrophages [31-34]. These data indicate that immune cells become immunologically tolerant after excessive inflammatory triggers, which can be induced by exercise. This physiological response is likely important to limit excessive inflammation, but might negatively host defence mechanisms (Figure 1, left lower pannel).

#### Immune dampening induced by moderate regular exercise

Regular moderate aerobic exercise has been suggested to have anti-inflammatory effects [1,3-5]. Furthermore, it limits immune activation induced by a single exercise session [29,35]. Regular moderate exercise is associated with reduced circulating numbers of CD16^+^ (inflammatory) monocytes, expression of TLR-4 in monocytes and increased circulating numbers of anti-inflammatory regulatory T cells (Tregs) [3]. In addition, Treg-associated cytokine production was enhanced, whereas production of pro-inflammatory cytokines by immune cells, visceral fat tissue and skeletal muscles was reduced [1,3,7-9,29]. These observations together suggest that regular moderate exercise reduces a pro-inflammatory state.

In conclusion, regular moderate exercise induces immune dampening. Immune cells, visceral fat and skeletal muscles adapt to exercise stimuli and produce lower amounts of pro-inflammatory modulators and increase production of anti-inflammatory products. Furthermore, circulating immune cells expressing lower levels of activation markers and immune cells which are associated with immune tolerance, such as Tregs, increase. These adaptations likely play a role in limiting clinical expression of diseases associated with inflammation (Figure 1, right panel).

#### Exercise and infection susceptibility

Besides the anti-inflammatory effects of regular moderate exercise, exercise also associates with upper respiratory tract infection (URTI) frequency and possibly other infectious diseases [4]. Although only limited data have been published, moderate regular aerobic exercise appears to reduce infection susceptibility in animals [36-39] and humans [40]. In contrast to moderate (regular) exercise, vigorous (regular) exercise is associated with an increased URTI susceptibility [4]. In animals it was shown that a period of moderate regular exercise reduces viral load, inflammation, morbidity and mortality upon a viral infection [36-39]. Recently, a large longitudinal cohort study in 1002 adults showed that during winter time the number of days with URTI and the symptom severity score were significantly lower in adults with a higher physical fitness level or exercise frequency, compared to adults with a low physical fitness level or exercise frequency [40].

Since salivary IgA levels increase upon a single exercise session and relate to URTI frequency [4], it can be speculated that changes in URTI frequency induced by regular moderate exercise are related to changes in sIgA levels. However the causality of this association requires further investigation. It also remains unclear whether exercise-associated URTIs are of infectious or inflammatory origin. Previous data showed that in subjects with exercise-associated URTI often no pathogens were found, supporting that exercise-related URTI can be caused by mechanically-induced inflammatory, rather than infectious, pathways [4].

Altogether, these results suggest that regular moderate exercise reduces infection susceptibility compared to high intensity exercise or being sedentary. It remains difficult to establish whether upper respiratory tract symptoms are of infectious or inflammatory origin. The impact of exercise on immune defence mechanisms against pathogens requires further investigation.

#### Molecular pathways that control exercise-induced inflammatory responses

Various pathways that are activated upon exercise to control the metabolic demand also have immune regulatory functions [1,41-44]. Exercise is associated with activation of the sympathetic nervous system and the hypothalamic-pituitary-adrenal (HPA) axis, which results in release of pleiotropic mediators such as catecholamines, glucocorticoids, cytokines and chemokines. Collectively, these mediators regulate the changing metabolic demand elicited by exercise [45].

All lymphoid and myeloid cells express receptors for catecholamines and glucocorticoids [43,44]. Induced cellular responses depend on the receptor repertoire expressed on a specific cell and the magnitude and duration of the catecholamine and glucocorticoids release [45]. Catecholamines modulate leukocyte gene transcription via stimulation of β-adrenergic receptors [44], pro-inflammatory cytokine genes expression (e.g. IL-1β, IL-6 and TNF-α) via activation of transcription factors such as CREB, GATA and nuclear factor kappa B (NF-κB) [44]. Furthermore, adrenergic stimulation leads to redistribution of innate immune cells between blood and tissues [44]. However, excessive stimulation of the HPA-axis or sympathetic nerve system, as observed by chronic stress, has been found to be immunosuppressive and lead to decreased immune effector functions and increased susceptibility to viral infection, prolonged wound healing and/ or decreased antibody production after vaccination [3,43]. Such conditions may also be evoked by high intensity exercise.

In general, glucocorticoid signalling suppresses inflammatory responses. Glucocorticoids can lead to a reduction in immune cell activation markers and lymphocyte proliferation [3,43,44]. Molecules involved in pathogen recognition (e.g. TLRs), antigen presentation (e.g. MHC-II) and adhesion (e.g. intracellular adhesion molecule 1) are down-regulated [43,44]. Furthermore, pro-inflammatory cytokine expression is reduced via inhibition of NF-κB and AP-1 activity [46]. Therefore, reciprocal regulation of NF-κB by catecholamines and glucocorticoids may play a crucial role in defining exercise-related immune responses. Glucocorticoids may restore the immune balance after a single exercise session via suppression of NF-κB activity, whereas catecholamines may activate the immune system during exercise via enhancing NF-κB activity (Figure 1, left upper and lower panel).

Peroxisome proliferator-activated receptor gamma co-activator 1 α(PGC-1α) is one of the key regulators of energy metabolism during exercise [1,41,47]. PGC-1α regulates genes involved in mitochondrial biogenesis, mitochondrial oxidative metabolism, gluconeogenesis and GLUT-4 expression in skeletal muscle [41]. Its expression is directly induced by an exercise-mediated increase of intracellular calcium levels [41,48] and quickly returns to baseline levels after cessation of the exercise session (Figure 1, left upper and lower panel) [1]. Regular exercise induces a permanent increase in skeletal muscle PGC1α levels (Figure 1, right panel) [1], which lead to differentiation of myocytes towards a more oxidative phenotype [41]. Additionally, PGC-1α has anti-inflammatory potential by negatively regulating NF-kB activity, which induces transcription of pro-inflammatory cytokines as has been demonstrated for IL-1β, IL-6 and TNFα [42,49]. Animal models lacking PGC-1α in skeletal muscles have a lower endurance exercise capacity [1,41] and an increased transcriptional induction of inflammatory genes [1]. Moreover, activation of peroxisome proliferator activated receptor γ (PPARγ) in monocytes, which is a down-stream target of PGC-1α, skews these cells into alternative M2 macrophages [49]. M2 macrophages produce cytokines, such as IL-10, transforming growth factor β (TGF-β) and IL-1 receptor antagonist (IL-1ra), which can dampen inflammation, whereas classical M1 macrophages have the capabilities to kill micro-organisms and produce pro-inflammatory cytokines [49]. The enhanced activity of the PGC-1α pathway, induced by regular exercise, may thus lead to a higher threshold for immune activation by negatively regulating NF-κB activity. These immune regulatory functions might also contribute to the anti-inflammatory effect of regular moderate exercise.

### Cystic fibrosis

#### Inflammation in patients with cystic fibrosis

Currently, chronic pulmonary infections are the main contributor to mortality and morbidity in patients with CF. These infections are caused by multiple micro-organisms including bacteria, fungi, and viruses, which lead to chronic inflammation and progressive pulmonary disease [11-13]. The most common bacterial pathogen infecting the lungs is Pseudomonas *aeruginosa* (P. *aeruginosa*), which ultimately infects up to 85% of CF patients [11-13]. Current literature suggests that chronic hyper-inflammatory responses in patients with CF are caused by multiple factors. Continuous stimulation of the immune system, due to impaired clearance of pathogens, and reduced anti-bactericidal capacities of CFTR-deficient immune cells, both contribute to sustained and severe inflammation in patients with CF [50,51].

When patients are clinically stable, an inflammatory profile is still observed. Increased levels of the pro-inflammatory interleukins (IL) IL1-β, IL-6, TNF-α and IL-8 are measured in peripheral blood and bronchoalveolar lavage fluid, whereas levels of the anti-inflammatory cytokine IL-10 are decreased [50,51]. These changes in cytokine levels are comparable to other chronic diseases, such as diabetes, obesity and atherosclerosis, which are characterized by low-grade inflammation and also show elevated levels of the cytokines IL-6, TNF-α and C-reactive protein (CRP) [3].

CFTR is an important regulator of cellular inflammatory homeostasis, and its absence has been found to be associated with increased NF-kB and decreased PPARγ activity [52]. This leads to chronic inflammation and excessive inflammatory responses to inflammatory stimuli in patients with CF [52]. Since exercise-induced PGC-1α may limit NF-kB activity [42,49], and increase PPAR-γ activity, regular training programmes in CF may restore the aberrations in NF-kB and PPAR-γ levels.

#### Exercise in cystic fibrosis disease

Patients with CF suffer from exercise intolerance and skeletal muscle atrophy. Physical fitness declines longitudinally during adolescence, resulting in a decreased exercise capacity of 20% at the age of 18 years old [20]. Exercise intolerance in patients with CF is associated with reduced pulmonary function [14-20], nutritional status [14-17,20], daily physical activity levels [53,54] chronic infection [20] and inflammation [20]. Chronic exposure to circulating pro-inflammatory mediators, such as TNF-α, IL-6, CRP and sphingomyelinase, has been related to muscle weakness in chronic inflammatory diseases [55]. TNF-α most strongly associates with muscle dysfunction, which has been attributed to a TNF-α induced loss of muscle mass and force [55]. Inflammatory markers such as CRP and total IgG levels also negatively associated with exercise capacity in patients with CF [20,56]. In adolescent patients with CF colonization with *P. aeruginosa* resulted in an exercise capacity decline of 4.60% [20].

Recently, it has been shown that CFTR is expressed at the sarcoplasmic reticulum of skeletal muscle and might contribute directly to exercise intolerance and muscle atrophy [57,58]. This may explain why CFTR^−/−^ mice are more vulnerable to muscle wasting and produce more myokines upon an infection with P. *aeruginosa* compared to CFTR^+/+^ mice [57]. In addition, CFTR was found to modulate skeletal muscle calcium homeostasis, musclar tone and metabolic recovery [59]. Furthermore, CFTR is important for ATP release by skeletal muscle upon reduction of intracellular pH in rats [60]. These findings indicate a direct role for CFTR in skeletal muscle, however CFTR genotype and physical fitness associated inconsistently in subjects with CF [20,61,62]. The exact role of CFTR in skeletal muscle therefore requires further research.

Whether exercise can affect CFTR expression or CFTR function in skeletal muscle is unknown, but it has been shown that the nasal epithelial sodium channel (ENaC) is inhibited during a single exercise session in healthy controls and patients with CF, whereas nasal chloride conductance did not change [63,64]. Inhibition of ENaC might facilitate mucus hydration by increasing mucus sodium levels and may therefore improve mucociliary clearance in patients with CF [63,64]. An inhaled radio-labelled aerosol randomized controlled study indeed showed that 20 minutes of exercise at 60% of maximal oxygen consumption resulted in an enhanced sputum clearance of 4% from the whole lung, 5% from the intermediate airways and 8% from the periphery, which was assessed by comparing the reduction in radioactivity in a pre-set time, pre- and post-exercise [65]. This may be related to an increased activity of ENaC, but it has also been suggested that mucus clearance is enhanced mechanically. Increased ventilation, shear forces and body movements would facilitate movement of mucus from the lung periphery to the oropharynx [66].

Patients with CF are encouraged to engaged in regular exercise, since the exercise capacity has been identified as an independent predictor of morbidity and mortality [20,67]. However, whether regular exercise can decrease morbidity and mortality in patients with CF remains controversial. Limited studies are available and results are inconsistent [21,68], which is probably due to the different exercise training programs used. Furthermore, high variability in adherence to the prescribed exercises, which has been shown to be low particularly in adolescents with CF [65], may also contribute to variable results.

Several studies, of which a panel was extensively reviewed in a Cochrane [21] and systematic review [68], showed that physical exercise training can improve exercise capacity, strength, quality of life and pulmonary function by enhancing mucociliary clearance and reducing residual volume in adults and children with CF [21,68-72]. Maximal oxygen consumption improved with 8.53 ml.kg^-1^.min^-1^ upon regular aerobic exercise in one randomized controlled trial [73], whereas another randomized controlled trial with 3-years follow-up did not find any differences between the exercise and non-exercise group [74]. Inconsistent results were found for pulmonary function as well, varying from no effect of regular exercise [73,75] to a slower annual decline in pulmonary function during 3 years follow-up in the exercise compared to non-exercise group [74].

Although exercise training induced inconsistent responses with regard to physical fitness and pulmonary function in patients with CF, individualized exercise training prescriptions in patients with CF, based on patient characteristics, such as baseline pulmonary function, exercise capacity, habitual physical activity levels, inflammation and infection status, would maybe helpful to establish favourable exercise-training induced effects. Although, it may also be possible that, at a certain point, disease deterioration may hamper patients with CF to improve from regular exercise.

#### Inflammatory responses to exercise in patients with cystic fibrosis

**Figure 2:**
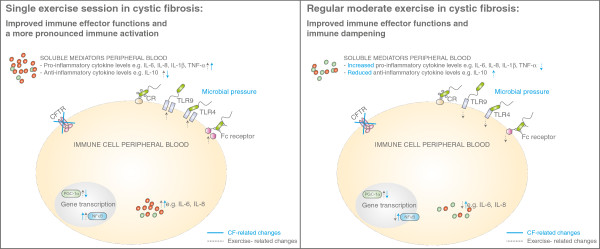
**Effect of exercise in inflammation in patients with cystic fibrosis, possible mechanisms.** It can be hypothesized that a single exercise session in patients with cystic fibrosis leads to a more pronounced immune activation, since both a single exercise session and cystic fibrosis lead to immune activation. A single exercise session in healthy subjects also leads to improved immune effector functions, therefore improved anti-bacterial immunity induced by exercise in patients with CF may also be hypothesized (left). Regular moderate exercise results in immune dampening and improved immune effector functions, which may therefore reduce chronic inflammation in CF and improve anti-bacterial defence mechanisms.

A single exercise session also leads to immune activation in patients suffering from chronic inflammatory diseases (e.g. diabetes mellitus, chronic obstructive pulmonary disease), however it elicits an aggravated inflammatory response compared to healthy individuals [6], which may suggest that exercise above a certain threshold may also aggravate symptoms in these patients. Additionally, levels of inflammatory markers (e.g. IL-6) and leukocyte levels remained elevated longer compared to healthy individuals [6]. Only a few studies examined the effect of exercise on inflammation in patients with CF. These showed that a single exercise session caused elevations in leukocyte subsets in children with CF similar to those found in healthy children [76,77]. However, after a single exercise session higher TNF-α [78], TNF-α soluble receptor I [79], IL-6 [78,79], and IL-6 soluble receptor [79] levels were measured in children [78] and adults [79] with CF compared to healthy individuals, which remained higher for a longer period as well [78,79]. These data suggest that a single exercise session in CF results in recruitment of cells with an activated phenotype, more predominantly than in healthy subjects (Figure 2, left panel).

Effect of single and regular exercise interventions in patients with other chronic inflammatory diseases (e.g. multiple sclerosis, chronic obstructive pulmonary disease, chronic heart failure, rheumatoid arthritis) have been reviewed. Ploeger et al. reported that a single exercise session might elicit inflammation, whereas regular exercise might dampen inflammation [6]. However, effects appeared to be dependent on the type of disease, disease severity and intensity and duration of the exercise intervention used [6].

Comparable to what is observed in healthy individuals, regular aerobic exercise has also been associated with reduced inflammatory conditions in patients with obesity, atherosclerosis, chronic heart failure, and coronary heart disease [1,3,4,6-10]. However contradictory results, e.g. in patients with multiple sclerosis, were also observed [6]. Inconsistency of results is probably related to the type of disease, disease severity and intensity, and the duration of the exercise intervention used. In a mouse model of allergic pulmonary inflammation it was shown that regular exercise could dampen inflammation. Reduced levels of eosinophils, the murine IL-8 homologue (CXCL1; KC), IL-4 and IL-5 in the pulmonary tissue and IgE in serum were measured [80,81].

In patients with CF only very few data are available describing the effects of regular aerobic exercise on inflammation [82,83]. A short-term regular exercise program in clinically stable female children with CF had no effect on pulmonary inflammation markers as indicated by total cell numbers in sputum and cytokine analysis in exhaled breath condensates [82]. However, another short-term regular exercise study showed that neutrophilic inflammation was reduced, indicated by reduced expression of CD11b (complement receptor 3), CD13 (aminopeptidase N), CD32 (low-affinity Fc γ chain receptor II), and CD35 (complement receptor 1) [83]. It remains unknown whether regular aerobic exercise has an effect on bacterial infection susceptibility in patients with CF.

Immune modulation in CF aims to prevent immune-mediated damage to pulmonary tissue whilst retaining or enhancing anti-microbial activity. Long-term exercise-induced effects have been proposed to decrease viral infection susceptibility in healthy individuals and animal models when exercise training is of moderate duration and intensity (Figure 2, right panel) [4]. However, moderate exercise programs in patients with CF may induce a physiological immune response comparable to high intensity training, due to the chronic inflammatory status in patients with CF. This may be different for individuals, and it may therefore be important to carefully monitor exercise programs for CF patients.

Furthermore, effects of regular moderate exercise on immune cell effector function and subsequent infection susceptibility remain unknown for these patients and needs to be addressed in long-term studies. Whether exercise modulates immune parameters and immune pathways that are modified in CF, such as NF-kB and PPARγ, should be investigated, which may be useful to give a more precise exercise prescription in patients with CF.

## Conclusion

Patients with CF suffer from severe and chronic pulmonary infections and inflammation, leading to obstructive and restrictive pulmonary disease, exercise intolerance and muscle cachexia. Regular exercise has beneficial effects in patients with CF with regard to preservation or improvement of pulmonary function, exercise capacity, muscle strength, quality of life, morbidity and mortality [21,68]. There is increasing evidence that exercise can modulate immune function in healthy persons and patients suffering from chronic inflammatory diseases, in an exercise-intensity-dependent fashion, of which patients with CF may also benefit.

Immune modulation in CF aims to prevent immune-mediated damage to pulmonary tissue whilst retaining or enhancing anti-microbial activity. In healthy individuals, regular moderate aerobic exercise has been shown to reduce inflammation and viral infection frequency. However, exercise of moderate intensity, established in healthy individuals, might exert different inflammatory responses in patients with chronic inflammatory conditions or when performed during active disease (e.g. pulmonary exacerbation). Physical fitness levels are often reduced in patients with a chronic disease. Since the intensity of an exercise depends on an individual’s previous exercise experience and relative level of fitness, exercise prescriptions should maybe be different for people with a chronic disease. Furthermore, exercise might induce differential inflammatory responses in subjects suffering from chronic infection and/or inflammation. Prescribed moderate exercise should therefore be individually assessed with regard to intensity, intervals, volume and timing (e.g. disease status).

Published data already showed enhanced immune activation upon a single exercise session and a slower resolution phase post-exercise in patients with CF. These data suggest that a single exercise session may aggravate already existing inflammation and should therefore perhaps be discouraged during periods of active infection in patients with CF (e.g. pulmonary exacerbation). This should be investigated by a randomized controlled trial in patients with CF suffering from a pulmonary exacerbation by monitoring disease duration, sickness severity, inflammatory markers and immune cell function.

In patients with CF, regular moderate aerobic exercise may reduce pro-inflammation by increasing PGC1α activity that limits NF-kB and enhances PPARγ function. Furthermore, regular moderate aerobic exercise affects infection susceptibility in healthy individuals. Although, it remains unclear whether alterations of immune cell function are the causal link between exercise-induced changes in infection rate, these changes would be beneficial for patients with CF as well. However, inflammatory responses to regular aerobic exercise remain to be firmly established in CF and other comparable inflammatory diseases.

In summary, more evidence is required to firmly establish which immunological parameters and pathways are regulated by (regular) exercise in patients with CF, how this relates to responses in healthy individuals, and how this will impact infection susceptibility. Better understanding of these mechanisms will be crucial for identifying patient-specific parameters that can be used to tailor individual exercise programs to optimize exercise-induced immune modulation.

## Abbreviations

CF: Cystic fibrosis; CFTR: Cystic fibrosis transmembrane conductance regulator; ENaC: Epithelial sodium channel; HLA: Human leukocyte antigens; HPA: Hypothalamic-pituitary-adrenal; IgGs: Immunoglobulins; IL: Interleukin; IL-1ra: Interleukin 1 receptor antagonist; LPS: Lipopolysaccharide; NF-κB: Nuclear factor-κB; P. aeruginosa: Pseudomonas *aeruginosa*; ROS: Reactive oxygen species; Tregs: Regulatory T cells; sIgA: Salivary immunoglobulin A; TLR: Toll-like receptors; TGF-β: Transforming growth factor-β; TNF-α: Tumour necrosis factor alpha; URTI: Upper respiratory tract infection; PGC-1α: Peroxisome proliferator-activated receptor gamma co-activator 1 α.

## Competing interests

“All authors have completed the Unified Competing Interest form and declare (1) financial support for the submitted work from The Dutch Cystic Fibrosis Foundation (NCFS); (2) CvdE has received research grants from Grünenthal and Glaxo Smith Kline; The other authors declare no interests under (2). (3) No spouses, partners, or children with relationships with commercial entities that might have an interest in the submitted work; (4) No non-financial interests that may be relevant to the submitted work.

## Authors’ contributions

The guarantor of this study is PvdW who is responsible for the integrity of the work as a whole, from conception and design to conduct of the review and writing of the manuscript. PvdW, HA, CvdE and JB designed the review. PvdW, HA, CvdE en JB wrote the manuscript. All authors reviewed and approved the final version.
